# Modern Gas Cooking Stoves

**Published:** 1908-05-09

**Authors:** 


### MODERN GAS COOKING STOVES.

Although the use of gas stoves for cooking is steadily growing in public favour, there are still people who believe that cooking by gas is malodorous and unhealthy, and also expensive. A visit to the show-rooms of the Richmond Company at 132 Queen Victoria Street, E.C., would greatly enlighten such prejudiced minds.

The company show there stoves large and small, from those suitable for a private family to those that meet the needs of a large institution, and the convenience and completeness of these would be apparent to anyone. The advantages of cooking by gas are its cleanliness and economy in labour and cooking materials.

At the present price of gas the difference of prime cost between it and raw fuel is very trifling. If the other factors are taken into consideration, the cost is probably no greater than in cooking with coal, and the convenience is immensely superior.

The Richmond roasters are strongly made, arranged so as to get the utmost possible value from the gas used, well ventilated, and, as they are enamelled inside, can be kept thoroughly clean. The vegetable-steamers are planned so as to secure equal heat for everything placed within them, and are of different sizes, the largest of which is ample for the needs of even a large institution. The heat that escapes from above the stove may be utilised for a hot-closet for warming plates.

The Company are the makers of an admirable fish-fryer, one of which has been recently installed at a large asylum and which is capable of frying 15 cwt. of fish at a time.

One of the new stoves is a suite of boilers enclosed in one range for the making of soup or anything that requires long and slow cooking. These have recently been ordered for the Municipality of Milan for meals for school-children, and it has been found that it is possible by means of them to cook meals for 500 children with less than 600 feet of gas, or an expenditure, taking the price of gas in London, of about 1s. 3d. Even where gas is dearer, the cost of cooking would be less than that of coal, and the boiler should be of great value in a children's hospital or wherever soup, puddings, and porridge are required. The beef-tea boilers and stock-pots are admirably constructed.

